# Deficits in blood culture collection in the emergency department if sepsis is suspected: results of a retrospective cohort study

**DOI:** 10.1007/s15010-024-02197-x

**Published:** 2024-03-05

**Authors:** Charlotte Berninghausen, Frank Schwab, Alexander Gropmann, Bernd A. Leidel, Rajan Somasundaram, Lydia Hottenbacher, Petra Gastmeier, Sonja Hansen

**Affiliations:** 1 Department of Cardiology and Intensive Care, Vivantes Wenckebach Klinikum, Berlin, Germany; 2 Department of Emergency Medicine, Vivantes Auguste-Viktoria Klinikum and Vivantes Wenckebach Klinikum, Berlin, Germany; 3 Institute of Hygiene and Environmental Medicine, Charité–Universitätsmedizin Berlin, corporate member of Freie Universität Berlin, Humboldt-Universität zu Berlin, and Berlin Institute of Health, Berlin, Germany; 4 Department of Emergency Medicine, Charité–Universitätsmedizin Berlin, corporate member of Freie Universität Berlin, Humboldt-Universität zu Berlin, and Berlin Institute of Health, Campus Benjamin Franklin, Berlin, Germany

**Keywords:** Blood cultures, Emergency department, Sepsis, Diagnostic stewardship, Rational antibiotic use

## Abstract

**Purpose:**

Blood cultures (BCs) are key for pathogen detection in septic patients. We investigated the extent to which sampling was performed and what factors were associated with the absence of general or inadequate BC sampling.

**Methods:**

We conducted a retrospective cohort study of hospitalized patients with sepsis admitted to one of three EDs in 2018. Primary outcome was the extent of general BC collection of at least 1 set. Secondary outcome was the extent of adequate BC sampling, defined as ≥ 2 sets before antibiotic therapy (AT). Multivariable logistic regression analysis was performed to identify factors associated with deficits in both outcomes.

**Results:**

1143 patients were analyzed. BCs were collected from 946 patients. Single BCs were taken from 520 patients, ≥ 2 sets from 426 patients. Overall, ≥ 2 BCs were taken from 349 patients before AT. BC sampling before AT occurred significantly more frequently when ≥ 2 BC sets were taken rather than a single one (81.9%, versus 68.4%, *p* < 0.001) and this also led to the highest pathogen detection rate in our cohort (65.6%). A body temperature of ≥ 38 °C was the a supporting factor for general and adequate BC collection in all three EDs. Retrospective analysis of 533 patients showed that the qSOFA score had no influence on general or adequate BC collection.

**Conclusion:**

Data on everyday clinical practice in the pre-analytical phase of microbiological diagnostics shows considerable deficits and indicates the need for more implementation of best practice. The variations identified in BC sampling between EDs should be further investigated.

**Supplementary Information:**

The online version contains supplementary material available at 10.1007/s15010-024-02197-x.

## Introduction

There is a significant association of sepsis with morbidity and mortality [1]. Numerous deaths resulting from sepsis could be prevented by targeted and adequate antibiotic therapy (AT) [2]. Identifying the causative pathogen is key to ensuring adequate AT. Blood cultures (BCs) continue to be the gold standard for detecting causative pathogens in patients with sepsis [3]. In 2017, the Surviving Sepsis Campaign (SSC) published guidelines to assist clinicians in identifying and treating septic patients. One of the cornerstones of these guidelines is appropriate diagnostics, in particular BC diagnostics [4]. Once sepsis is suspected, a minimum of two sets of BCs should be obtained immediately. Although recently updated international guidelines do not refer explicitly to the necessity of BCs [5], data shows that it is nonetheless essential that at least two sets of BCs be taken from patients before the administration of AT [6, 7]. This recommendation continues to be part of current German guidelines [8]. Indeed, Collazos-Blanco et al. suggest that three sets should be obtained per patient to ensure a greater probability of identifying the causative pathogen [9].

Numerous studies have investigated the implementation of sepsis guidelines and recommendations [10–13]. However, although these studies have examined whether BCs were obtained, they did not take into consideration the timing in relation to AT administration or the number of sets taken per patient.

Because emergency departments (EDs) are most frequently involved in the early diagnosis and treatment of patients with community-onset sepsis [10], we describe the results of a retrospective analysis of BC sampling in patients with suspected sepsis in three German EDs with a focus on (1) BC sampling in general and (2) an adequate BC collection of two BC sets before AT administration. Furthermore, we analyze factors associated with gaps in general and adequate BC collection.

## Methods

### Study design and setting

In this retrospective cohort study, we analyzed the BC sampling in EDs of hospitalized patients with a hospital discharge diagnosis of “sepsis” based on ICD-10 (International Statistical Classification of Diseases and Related Health Conditions) System A40-A41 [14]. Data was obtained in three EDs (A, B and C) of acute care hospitals: Hospital A is a primary care hospital with 443 beds and 15,809 ED patient visits in 2018; hospital B is a secondary care hospital with 692 beds and 34,368 ED patient visits; hospital C is a tertiary care hospital with 830 beds and 44,782 ED patient visits.

### Study population

Included were all inpatients ≥ 18 years of age admitted to hospitals A–C via the ED and who were discharged between 01.01.2018 and 31.12.2018 with a diagnosis of “sepsis” [14]. Only patients who had received emergency care in EDs A–C with standardized documentation were included.

### Exclusion criteria

Patients were excluded from the analysis (1) if patients’ admission had been planned prior to their visit to the ED; (2) if they had been transferred from another hospital; (3) if they were admitted directly to the intensive care unit; or (4) if they were diagnosed with hospital-acquired sepsis. Hospital-acquired sepsis was diagnosed by identifying signs and symptoms of sepsis that first appeared on day 3 or later.

### Outcome

The primary outcome was the extent to which a BC consisting of at least one set was generally taken during the initial treatment of a patient in the ED. The secondary outcome was the extent of adequate BC collection. Adequate BC sampling was defined as a BC consisting of at least two sets per patient prior to the administration of AT, each set containing one anaerobe and one aerobe sample. Gaps in primary and secondary outcomes were further analyzed in relation to their association with patient-based and organizational factors.

In addition, pathogen detection rates were analyzed for patients receiving 1 or more BC sets before or after AT administration.

### Data source and collection

Eligible patients were reviewed and analyzed for inclusion and exclusion criteria. The following data was obtained from the patient charts: patient characteristics and routine clinical data such as information on vital signs, possible immunosuppression, relevant comorbidities and medication, the presumed (source of) infection, and the chronological course of the taking of BCs and the administration of AT. Furthermore, data from the microbiological laboratory was evaluated in order to obtain as much information on BC sampling and the exact number of sets taken per patient. Data was entered into the study database using the online survey tool Lime survey, version 2.0.

### Data analysis

In the descriptive analysis we specified number and percent for categorical parameters and median and interquartile range (IQR) for continuous parameters. Differences were tested using the chi-square test or the Wilcoxon rank-sum test. A multivariable logistic regression analysis was performed to examine factors associated with deficits in primary and secondary outcomes. All patient characteristics as well as clinical and laboratory parameters documented in the ED were taken into consideration in the analysis. Parameters not documented were categorized as “no information” and considered as a separate category. The multivariable model was calculated for (a) all three EDs and (b) individual EDs with stepwise forward variable selection where the significance level was set at <  = 0.05 to include a parameter in the model. As part of a sensitivity analysis for data of all three EDs, we also considered the parameter based on the physicians’ estimate of the suspected focus of the infection in the model. All analyses were exploratory in nature and were performed with SAS (version 9.4) and SPSS (version 25).

## Results

Overall, data from 1143 patients was included in the analysis. As shown in Table 1, BCs were taken from 946 patients (83%), most often one set (*n* = 520; 46%). Two or more sets were taken from 426 patients (37%). The overall rate of BCs taken did not differ among EDs A, B, and C (82, 82. and 83%, respectively), while the number of sets per patient varied significantly between the EDs with ≥ 2 BC sets for 30% of patients in ED C to 78% in ED A (*p* < 0.001). Significant differences were also seen in the AT administration documented. With 801 patients (70%) documented overall, the highest rate of documentation of AT administration occurred in ED C (75%), followed by ED A (71%) and ED B (62%). Overall, 349 patients (31%) had a documented adequate sampling of ≥ 2 BC sets prior to the administration of AT. Patients received antibiotics significantly more frequently after BC collection when two or more sets were taken rather than a single BC (81.9%, versus 68.4%, *p* < 0.001).

**Table 1:** Number of blood culture sets per patient with suspected sepsis in the emergency department (*n* = 1143 patients)

	ED A *n* (%)	ED B *n* (%)	ED C *n* (%)	All *n* (%)	*P* value
All patients	112 (100)	376 (100)	655 (100)	1143 (100)	
No BC taken	20 (17.9)	68 (18.1)	109 (16.6)	197 (17.2)	0.826
1 BC set taken	5 (4.5)	165 (43.9)	350 (53.4)	520 (45.5)	< 0.001
1 BC set taken prior to AT administration	4/5 (80)	121/165 (73.3)	230/350 (65.7)	355/520 (68.3)	0.190
≥ 2 BC sets taken	87 (77.7)	143 (38)	196 (29.9)	426 (37.2)	< 0.001
≥ 2 BC sets taken prior to AT administration	72/87 (82.8)	119/143 (83.2)	158/196 (80.6)	349/426 (81.9)	0.807

*ED* Emergency department, *BC* Blood culture, *AT* Antibiotic therapy

**Fig. 1:**
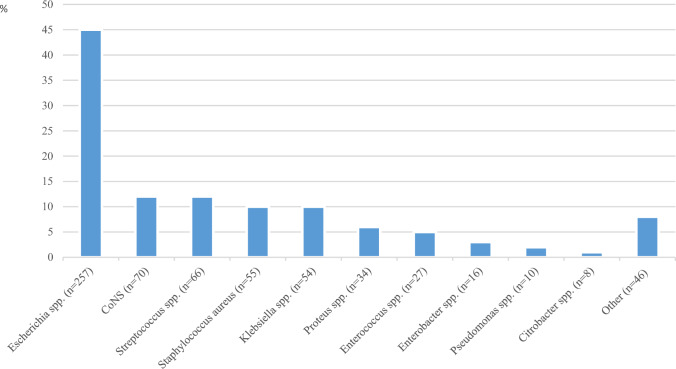
Pathogens detected in septic patients with positive blood cultures (*n* = 570 patients). Spp: Species; CoNS: Coagulase-negative staphylococci; Other: other than mentioned

(6) The most frequent pathogens identified are shown in Fig. 1.

The median age of the patients was 75 (interquartile range (IQR) 65–81); they were predominantly male (59%). Forty-seven percent of patients were immunocompromised, whereby age and percentage of immunocompromised patients differed significantly between the 3 Eds (Supplement Table 1). All patients’ characteristics are stratified in accordance to general BC sampling and adequate BC collection in Table 2.

**Table 2:** General characteristics and comorbidities of patients stratified in accordance to general sampling of blood cultures (BC) and adequate BC collection (*n* = 1143)

Parameter	Category	All	No BC	> = 1 BC	*P* value	Adequate BC collection^A^	No adequate BC collection	*P* value
		*n* (%)/median (IQR)				*n* (%)/median (IQR)		
Patients		1143 (100)	197 (100)	946 (100)		349 (100)	794 (100)	
ED	A	112 (9.8)	20 (10.2)	92 (9.7)	0.826	72 (20.6)	40 (5)	< 0.001
	B	376 (32.9)	68 (34.5)	308 (32.6)		119 (34.1)	257 (32.4)	
	C	655 (57.3)	109 (55.3)	546 (57.7)		158 (45.3)	497 (62.6)	
Age (years)		75 (65–81)	75 (66–81)	75 (65–81)	0.869	75 (64–81)	75 (65–81)	0.904
Age (years)	< 65	280 (24.5)	46 (23.4)	234 (24.7)	0.809	89 (25.5)	191 (24.1)	0.693
	65–74	271 (23.7)	44 (22.3)	227 (24)		75 (21.5)	196 (24.7)	
	75–84	412 (36)	77 (39.1)	335 (35.4)		130 (37.2)	282 (35.5)	
	≥ 85	180 (15.7)	30 (15.2)	150 (15.9)		55 (15.8)	125 (15.7)	
Gender	Male	676 (59.1)	108 (54.8)	568 (60)	0.351	225 (64.5)	451 (56.8)	0.044
	Female	466 (40.8)	89 (45.2)	377 (39.9)		124 (35.5)	342 (43.1)	
	Other	1 (0.1)	0 (0)	1 (0.1)		0 (0)	1 (0.1)	
Documented comorbidities^B^								
Chronic renal failure		199 (17.4)	34 (17.3)	165 (17.4)	0.951	61 (17.5)	138 (17.4)	0.968
Diabetes mellitus		299 (26.2)	50 (25.4)	249 (26.3)	0.785	103 (29.5)	196 (24.7)	0.087
Lymphoma		24 (2.1)	0 (0)	24 (2.5)	0.024	12 (3.4)	12 (1.5)	0.036
Leucemia		17 (1.5)	1 (0.5)	16 (1.7)	0.212	2 (0.6)	15 (1.9)	0.091
HIV/AIDS		23 (2)	2 (1)	21 (2.2)	0.273	13 (3.7)	10 (1.3)	0.006
Documented immunosupression^B^		542 (47.4)	80 (40.6)	462 (48.8)	0.035	190 (54.4)	352 (44.3)	0.002
Due to medication		155 (13.6)	17 (8.6)	138 (14.6)	0.053	55 (15.8)	100 (12.6)	< 0.001
Due to comorbidity		466 (40.8)	69 (35)	397 (42)	0.196	162 (46.4)	304 (38.3)	0.018

*BC* Blood culture, *IQR* Interquartile range

^A^Adequate BC collection in ED: ≥ 2 sets per patient prior to administration of antibiotic therapy

^B^Multiple answers possible

As summarized in Table 3, 46% of patients had a body temperature above 38 °C and 18% had systolic blood pressure < 100 mm Hg. A respiratory rate of > 20 breaths per minute was documented in 28% of patients.

**Table 3:** Clinical and laboratory findings in respect of organ dysfunction of patients with suspected sepsis stratified in accordance to general sampling of blood cultures (BC) and adequate BC collection (*n* = 1143)

Parameter	Category	All (*n*=1143) *n* (%)/median (IQR)	No BC (n=197) *n* (%)/media*n* (IQR)	>=1 BC (*n*=946) *n* (%) / median (IQR)	*P* value	Adequate BC collection^A^ (*n*=349)	No adequate BC collection (*n*=794)	*P* value
Heart or pulse rate (beats/min)	≥ 90	668 (58.4)	89 (45.2)	579 (61.2)	< 0.001	224 (64.2)	444 (55.9)	< 0.001
	< 90	296 (25.9)	52 (26.4)	244 (25.8)		101 (28.9)	195 (24.6)	
	No information	179 (15.7)	56 (28.4)	123 (13)		24 (6.9)	155 (19.5)	
Systolic blood pressure (mmHg)	≤ 100	228 (19.9)	37 (18.8)	191 (20.2)	< 0.001	83 (23.8)	145 (18.3)	< 0.001
	101 to < 140	481 (42.1)	61 (31)	420 (44.4)		158 (45.3)	323 (40.7)	
	≥ 140	260 (22.7)	44 (22.3)	216 (22.8)		87 (24.9)	173 (21.8)	
	No information	174 (15.2)	55 (27.9)	119 (12.6)		21 (6)	153 (19.3)	
Respiratory rate (breaths/min)	≥ 20	324 (28.3)	46 (23.4)	278 (29.4)	0.058	104 (29.8)	220 (27.7)	0.014
	< 20	330 (28.9)	52 (26.4)	278 (29.4)		117 (33.5)	213 (26.8)	
	No information	489 (42.8)	99 (50.3)	390 (41.2)		128 (36.7)	361 (45.5)	
Body temperature (°C)	≥ 38	522 (45.7)	33 (16.8)	489 (51.7)	< 0.001	190 (54.4)	332 (41.8)	< 0.001
	36° < 38	408 (35.7)	99 (50.3)	309 (32.7)		105 (30.1)	303 (38.2)	
	< 36	60 (5.2)	12 (6.1)	48 (5.1)		18 (5.2)	42 (5.3)	
	No information	153 (13.4)	53 (26.9)	100 (10.6)		36 (10.3)	117 (14.7)	
Suspected focus of infection	Respiratory tract	228 (19.9)	27 (13.7)	201 (21.2)	< 0.001	78 (22.3)	150 (19)	< 0.001
	Urinary tract	389 (34)	57 (28.9)	332 (35.1)		98 (28.1)	291 (37)	
	Intraabdominal	96 (8.4)	20 (10.2)	76 (8)		30 (9)	66 (8.3)	
	Skin and soft tissue	35 (3.1)	9 (4.6)	26 (2.7)		10 (3)	25 (3.1)	
	Intravascular device	11 (1)	2 (1)	9 (1)		6 (2)	5 (1)	
	Other	9 (0.8)	0 (0)	9 (1)		3 (1)	6 (1)	
	Unknown	239 (20.9)	27 (13.7)	212 (22.4)		104 (30)	135 (17)	
	No information	136 (11.9)	55 (27.9)	81 (8.6)		20 (5.7)	116 (15)	
Encephalopathy								
Disorientation	Yes	199 (17.4)	31 (15.7)	168 (17.8)	0.030	75 (21.5)	124 (15.6)	<0.001
	No	477 (41.7)	69 (35)	408 (43.1)		165 (47.3)	312 (39.3)	
	No information	467 (40.9)	97 (49.2)	370 (39.1)		109 (31.2)	358 (45.1)	
Altered mentation^B^	Yes	229 (20)	39 (19.8)	190 (20.1)	0.005	74 (21.2)	155 (19.5)	<0.001
	No	663 (58)	98 (49.7)	565 (59.7)		230 (65.9)	433 (54.5)	
	No information	251 (22)	60 (30.5)	191 (20.2)		45 (12.9)	206 (25.9)	
Respiratory dysfunction^C^	Yes	393 (34.4)	48 (24.4)	345 (36.5)	<0.001	136 (39)	257 (32.4)	0.001
	No	553 (48.4)	97 (49.2)	456 (48.2)		174 (49.9)	379 (47.7)	
	No information	197 (17.2)	52 (26.4)	145 (15.3)		39 (11.2)	158 (19.9)	
Renal dysfunction^D^	Yes	769 (67.3)	136 (69)	633 (66.9)	0.042	231 (66.2)	538 (67.8)	0.131
	No	343 (30)	51 (25.9)	292 (30.9)		113 (32.4)	230 (29)	
	No information	31 (2.7)	10 (5.1)	21 (2.2)		5 (1.4)	26 (3.3)	
Liver failure	Yes	166 (14.5)	19 (9.6)	147 (15.5)	<0.001	50 (14.3)	116 (14.6)	0.776
	No	618 (54.1)	95 (48.2)	523 (55.3)		194 (55.6)	424 (53.4)	
	No information	359 (31.4)	83 (42.1)	276 (29.2)		105 (30.1)	254 (32)	
Coagulation failure^F^	Yes	630 (55.1)	111 (56.3)	519 (54.9)	0.023	204 (58.5)	426 (53.7)	0.014
	No	451 (39.5)	68 (34.5)	383 (40.5)		136 (39)	315 (39.7)	
	No information	62 (5.4)	18 (9.1)	44 (4.7)		9 (2.6)	53 (6.7)	
Elevated INR^G^	Yes	494 (43.2)	99 (50.3)	395 (41.8)	0.003	146 (41.8)	348 (43.8)	0.003
	No	126 (11)	9 (4.6)	117 (12.4)		55 (15.8)	71 (8.9)	
	No information	523 (45.8)	89 (45.2)	434 (45.9)		148 (42.4)	375 (47.2)	
Thrombocytopenia^H^	Yes	266 (23.3)	29 (14.7)	237 (25.1)	<0.001	102 (29.2)	164 (20.7)	0.007
	No	361 (31.6)	81 (41.1)	280 (29.6)		101 (28.9)	260 (32.7)	
	No information	516 (45.1)	87 (44.2)	429 (45.3)		146 (41.8)	370 (46.6)	
Elevated lactate level^I^	Yes	512 (44.8)	92 (46.7)	420 (44.4)	<0.001	151 (43.3)	361 (45.5)	0.261
	No	352 (30.8)	40 (20.3)	312 (33)		119 (34.1)	233 (29.3)	
	No information	279 (24.4)	65 (33)	214 (22.6)		79 (22.6)	200 (25.2)	
Total leukocyte count	Leukocytosis^J^	682 (59.7)	120 (60.9)	562 (59.4)	<0.001	199 (57)	483 (60.8)	0.023
	Leukopenia^K^	98 (8.6)	9 (4.6)	89 (9.4)		41 (11.7)	57 (7.2)	
	Normal leukocyte count^L^	356 (31.1)	63 (32)	293 (31)		109 (31.2)	247 (31.1)	
	No information	7 (0.6)	5 (2.5)	2 (0.2)		0 (0)	7 (0.9)	
Documentation of all relevant qSOFA criteria ^M^	Yes	553 (48.4)	79 (40.1)	474 (50.1)	0.011	199 (57)	354 (45)	<0.001
	No	590 (51.6)	118 (59.9)	472 (49.9)		150 (43)	440 (55)	
qSOFA Score^N^	≥ 2	277 (50.1)	41 (51.9)	236 (49.8)	0.728	99 (49.7)	178 (50.3)	0.904
	< 2	276 (49.9)	38 (48.1)	238 (50.2)		100 (50.3)	176 (49.7)	

*BC* Blood culture, *IQR* Interquartile range, *GCS* Glasgow Coma Scale, *qSOFA* Quick Sequential Organ Failure Assessment

^A^Adequate BC collection in ED: ≥ 2 blood culture sets prior to administration of antibiotics

^B^Altered mentation: GCS (Glasgow Coma Scale) < 15

^C^Respiratory dysfunction: Oxygen saturation < 95%

^D^Renal dysfunction: Elevated serum creatinine level ≥ 1.3 mg/dl male, ≥ 1.1 mg/dl female

^E^Liver failure: Elevated bilirubin level ≥ 1.2 mg/dl

^F^Coagulation failure: INR > 1.25 and/or Thrombocytopenia < 150 × 109/L

^G^Pathological INR: > 1.25

^H^Thrombocytopenia: < 150 × 109/L

^I^Elevated lactate level: > 18.0 mg/dl

^J^Normal white blood count: 4–12 (× 109/L)

^K^Leukocytosis: ≥ 12 (× 109/L)

^L^Leukopenia: < 4 (× 109/L)

^M^Documentation of respiratory rate, systolic blood pressure and mental status (disorientation and/or altered mentation)

^N^Subanalysis of 553 Patients with all criteria of qSOFA documented

The analysis of 553 patients for whom all criteria of the qSOFA score were available showed that patients with a retrospectively determined qSOFA score >  = 2 received general or no BC collection to the same extent (49.8% versus 51.9%). This distribution was also evident in patients with a retrospectively determined qSOFA score of < 2 who did or did not receive adequate BC sampling (49.7% versus 50.3%).

### Multivariable logistic regression and sensitivity analysis

Multivariable logistic regression analysis for data from all three EDs showed a decreased likelihood of general BC collection in patients with a systolic blood pressure ≥ 140 mmHg and for whom data on mental alteration or systolic blood pressure was missing. Medically induced immunosuppression or a body temperature ≥ 38 °C were supporting factors for receiving BCs (Table 4).

**Table 4:** Results of (a) multivariable logistic regression analysis with outcome no blood culture sampling and (b) sensitivity analysis

Parameter	Category	OR	95% CI	*P* value
(a) Outcome “No BC collection”				
Altered mentation^A^	Yes	1.04	(0.68–1.61)	0.847
	No information	1.70	(1.14–2.55)	0.010
	No	1 = reference		
Body temperature (°C)	< 36	0.75	(0.38–1.49)	0.410
	≥ 38	0.19	(0.12–0.28)	< 0.001
	No information	1.20	(0.72–1.99)	0.488
	36 < 38	1 = reference		
Systolic blood pressure (mmHg)	< = 100	1.02	(0.64–1.61)	0.950
	> = 140	1.56	(1–2.43)	0.048
	No information	1.87	(1.1–3.19)	0.021
	> 100- < 140	1 = reference		
Medically induced immunosuppression	Yes	0.57	(0.33–0.99)	0.044
	No	1 = reference		
(b) Outcome “No BC collection”				
Sensitivity Analysis with presumed source of infection				
Altered mentation^A^	Yes	1.12	(0.71–1.76)	0.618
	No information	1.78	(1.18–2.7)	0.006
	No	1 = reference		
Body temperature (°C)	< 36	0.70	(0.34–1.43)	0.329
	≥ 38	0.20	(0.13–0.31)	< 0.001
	No information	1.10	(0.65–1.86)	0.726
	36 < 38	1 = reference		
Systolic blood pressure (mmHg)	< = 100	1.03	(0.65–1.66)	0.889
	> = 140	1.46	(0.92–2.29)	0.106
	No information	2.03	(1.17–3.51)	0.012
	> 100- < 140	1 = reference		
Medically induced immunosuppression	Yes	0.58	(0.33–1.01)	0.054
	No	1 = reference		
Suspected focus of infection: Intraabdominal	Yes	1.89	(1.07–3.33)	0.028
	No	1 = reference		
Suspected focus of infection: Skin and soft tissue	Yes	2.68	(1.15–6.24)	0.023
	No	1 = reference		
Suspected focus of infection: No information	Yes	3.65	(2.39–5.58)	< 0.001
	No	1 = reference		

*BC* Blood culture, *OR* Odds ratio, *CI* Confidence interval, *ED* Emergency department

^A^Altered mentation: Glasgow Coma Scale < 15

The likelihood of adequate BC collection with ≥ 2 BC sets before AT was lower for female patients, for patients with missing data on mental alteration or blood pressure, and in certain EDs.

Patients with a temperature ≥ 38 °C, systolic blood pressure ≤ 100 mm Hg, or documented immunosuppression had a higher chance that adequate BC collection would take place (Table 5).

**Table 5:** Results of (a) multivariable logistic regression analysis with outcome no adequate blood culture sampling and (b) sensitivity analysis

Parameter	Category	OR	95% CI	*P* value
(a) Outcome “No adequate BC sampling”^A^				
ED	B	3.03	(1.89–4.86)	< 0.001
	C	5.66	(3.62–8.85)	< 0.001
	A	1 = Reference		
Gender	Female	1.53	(1.16–2.03)	0.003
	Male	1 = Reference		
Altered mentation^B^	Yes	1.01	(0.71–1.43)	0.957
	No information	1.74	(1.19–2.56)	0.005
	No	1 = Reference		
Body temperature (°C)	< 36	0.83	(0.44–1.57)	0.572
	≥ 38	0.53	(0.39–0.72)	< 0.001
	No information	0.59	(0.35–1)	0.051
	36 < 38	1 = Reference		
Systolic blood pressure (mmHg)	< = 100	0.79	(0.55–1.13)	0.198
	> = 140	1.15	(0.81–1.62)	0.439
	No information	3.91	(2.19–6.99)	< 0.001
	> 100- < 140	1 = Reference		
Immunocompromisation	Yes	0.68	(0.52–0.9)	0.006
	No	1 = Reference		
(b) Outcome “No adequate BC sampling”^A^, Sensitivity analysis with presumed source of infection				
ED	B	3.09	(1.91–4.98)	< 0.001
	C	5.46	(3.48–8.57)	< 0.001
	A	1 = Reference		
Gender	Female	1.51	(1.14–2.01)	0.004
	Male	1 = Reference		
Altered mentation^B^	Yes	1.04	(0.73–1.48)	0.831
	No information	1.69	(1.15–2.49)	0.008
	No	1 = Reference		
Body temperature (°C)	< 36	0.84	(0.44–1.59)	0.583
	≥ 38	0.57	(0.42–0.78)	0.001
	No information	0.59	(0.34–1)	0.05
	36 < 38	1 = Reference		
Systolic blood pressure (mmHg)	< = 100	0.82	(0.57–1.18)	0.291
	> = 140	1,1	(0.78–1.56)	0.599
	No information	3.78	(2.11–6.77)	< 0.001
	> 100- < 140	1 = Reference		
Medically induced immunosuppression	Yes	0.72	(0.54–0.95)	0.019
	No	1 = Reference		
Suspected focus of infection: unknown	Yes	0.65	(0.47–0.9)	0.010
	No	1 = Reference		
Suspected focus of infection: not documented	Yes	2.09	(1.23–3.54)	0.006
	No	1 = Reference		

*BC* Blood culture, *OR* Odds ratio, *CI* Confidence interval, *ED* Emergency department

^A^Adequate BC collection in ED: ≥ 2 blood cultures prior to administration of antibiotics

^B^Altered mentation: Glasgow Coma Scale < 15

### Sensitivity analysis

For both outcomes, factors identified in the multivariate analysis were confirmed in the sensitivity analysis, which took into account the respectively presumed focus of the infection.

### Multivariable logistic regression for individual EDs

Additional multivariable logistic regression analyses for each individual ED identified a body temperature ≥ 38 °C as supporting factor for receiving general and adequate BC sampling in all 3 EDs. Factors, such as medically induced immunosuppression or missing data on systolic blood pressure, were only significant in individual EDs (Table 6).

**Table 6:** Results of multivariable logistic regression analysis for individual emergency departments A–C with outcome (a) no blood culture sampling and (b) no adequate blood culture sampling

Parameter	Category	ED 1			ED 2			ED 3		
		OR	95% CI	*P* value	OR	95% CI	*P* value	OR	95% CI	*P* value
(a) Outcome “No BC sampling”										
Altered mentation^A^	Yes	0.36	(0.07–2.04)	0.250	1.48	(0.65–3.4)	0.351	0.92	(0.52–1.64)	0.783
	No information	7.19	(0.89–58.15)	0.064	1.49	(0.75–2.96)	0.254	1.64	(0.95–2.81)	0.074
	No	1 = reference			1 = reference			1 = reference		
Body temperature (°C)	< 36	1.99	(0.28–14.18)	0.494	0.75	(0.14–4.1)	0.737	0.76	(0.33–1.76)	0.520
	≥ 38	0.14	(0.03–0.72)	0.019	0.32	(0.15–0.68)	0.003	0.12	(0.06–0.22)	< 0.001
	No information	11.53	(0.89–150.22)	0.062	1.21	(0.55–2.68)	0.635	1.43	(0.61–3.34)	0.410
	36–< 38	1 = reference			1 = reference			1 = reference		
Systolic blood pressure (mmHg)	< = 100	5.41	(1.08–27.17)	0.040	0.70	(0.22–2.21)	0.538	0.99	(0.56–1.74)	0.970
	> = 140	1.62	(0.34–7.79)	0.549	3.27	(1.38–7.73)	0.007	1.22	(0.67–2.23)	0.517
	No information	NE	NE	1.000	3.08	(1.27–7.48)	0.013	1.31	(0.54–3.14)	0.552
	> 100–< 140	1 = reference			1 = reference			1 = reference		
Medically induced immunosuppression	Yes	NE	NE	1.000	0.13	(0.02–1.01)	0.051	0.76	(0.41–1.39)	0.375
	No	1 = reference			1 = reference			1 = reference		
(b) Outcome “No adequate BC sampling”^B^										
Gender	Female	1.00	(0.4–2.48)	0.997	1.29	(0.81–2.08)	0.286	1.96	(1.31–2.93)	0.001
	Male	1 = reference			1 = reference			1 = reference		
Altered mentation^A^	Yes	0.85	(0.28–2.58)	0.777	1.16	(0.62–2.18)	0.641	0.87	(0.54–1.4)	0.560
	No information	1.53	(0.26–9.11)	0.643	2.57	(1.31–5.06)	0.006	1.41	(0.85–2.31)	0.181
	No	1 = reference			1 = reference			1 = reference		
Body temperature (°C)	< 36	0.57	(0.11–2.84)	0.493	0.51	(0.14–1.84)	0.302	1.33	(0.54–3.25)	0.537
	≥ 38	0.24	(0.09–0.6)	0.002	0.43	(0.23–0.8)	0.008	0.66	(0.44–0.98)	0.039
	No information	3.35	(0.31–36.55)	0.322	0.30	(0.14–0.64)	0.002	1.84	(0.56–6.09)	0.315
	36- < 38	1 = reference			1 = reference			1 = reference		
Systolic blood pressure (mmHg)	< = 100	1.60	(0.5–5.16)	0.428	0.66	(0.34–1.31)	0.240	0.80	(0.5–1.27)	0.346
	> = 140	1.26	(0.46–3.44)	0.658	1.01	(0.55–1.87)	0.978	1.17	(0.73–1.88)	0.519
	No information	NE	NE	1.000	2.92	(1.4–6.1)	0.004	5.80	(1.22–27.61)	0.027
	> 100- < 140	1 = reference			1 = reference			1 = reference		

*ED* Emergency department, *BC* Blood culture, *CI* Confidence interval, *NE* Not estimable

^A^Altered mentation: GCS (Glasgow Coma Scale) < 15

^B^Adequate BC collection: ≥ 2 blood culture sets prior to administration of antibiotics

## Discussion

In our retrospective study, we analyzed data from 1143 patients prior to their inpatient admission for suspected sepsis. This is a rather small proportion of a total 95,000 patient visits to the 3 participating EDs per year. However, the importance of adequate BC diagnostics in early treatment of sepsis warrants a precise consideration of BC sampling as an important part of AMS in healthcare [13].

Interestingly, at least one BC was taken from an equally high proportion of patients during initial care. This shows that the risk of sepsis was perceived as often in all three EDs. Only single BCs were obtained from the majority of patients, which indicates that a suspicion of sepsis was followed up with a BC during these patients’ initial care, although sampling was not in accordance with best practice. As described by Fabre et al. in a survey in the US, HCWs are often of the opinion that a single set of BCs is adequate for detecting bacteremia [15].

No BC at all was taken from almost 20% of patients. Whether this reflects an effort to avoid excessive BC diagnostics as described in the literature, remains unclear [16]. One common argument for a more limited use of BC diagnostics is to avoid unnecessary treatment if contamination of a culture has occurred. In emergency care, however, it can be assumed that the clinical picture takes precedence and that initial AT given is independent of the results of BC diagnostics, which are only available later. Therefore, we cannot fully support the approach of restricting the use of BC diagnostics, particularly in patients who show signs of infection and who are likely to require hospital admission. Of course, contamination of samples is a problem, and all healthcare departments—EDs included—should check the contamination rates of microbiological samples regularly and implement appropriate interventions when necessary. Rather than restricting the number of BCs taken, we believe the optimal means of preventing contamination is the strict aseptic sampling of at least two BC sets, each obtained from different peripheral sites [3]. The recommended number of at least two BC sets per patient was obtained only for a good third of all patients. This indicates that a sampling of two sets is possible during patient care in an ED. In addition, the high proportion of patients from whom at least two sets were taken prior to AT administration suggests that following this recommended time sequence is also feasible. Interestingly, the chronological order of BC collection and AT administration was significantly more in line with recommendations when ≥ 2 sets were taken rather than only a single BC. In addition, the recommended chronological order was associated with a higher pathogen detection rate, which was highest in patients receiving ≥ 2 sets before AT administration in our cohort.

More education and training on identification of patients for BC diagnostic and adequate BC sampling are needed [17] and obstacles and facilitators should be addressed during implementation. Our analyses of data from all three EDs and data from individual EDs show that BCs in general were more likely to be obtained generally and adequately if patients had an elevated body temperature. This result is in line with the findings of the survey by Fabre et al. in which HCWs acknowledged that febrile patients were more likely to yield positive BCs [15]. In addition, a German nationwide survey found that fever above 38.5 °C is a very strong clinical criterion for BC sampling [18]. But increased body temperature is not a specific sign of sepsis, especially in the case of elderly patients, who represent the largest segment of patients in our study [19]. Therefore, close attention to elevated temperature should be balanced by an awareness that a temperature below the cut-off level does not rule out infection. According to our data an immunocompromised state seems also to be a strong stimulus for initiating microbiological blood analysis in the ED. Although the clinical presentation of sepsis might differ depending on immune status [20], healthcare workers’ (HCW) awareness of adequate microbiological diagnostics may be increased by the fact that immunosuppressive medications increase the risk of sepsis [21, 22].

With the exception of elevated body temperature obstacles and facilitators identified in the analysis of all three EDs were not confirmed for individual EDs. This may be due to individual analyses’ smaller numbers and to potential differences between the three cohorts, which were selected by hospitals’ coding of sepsis [23]. Another factor could be the variations in the pre-analytical phase of BC diagnostics among the EDs.

In one ED a gender bias was associated with the outcome of no adequate BC sampling since adequate BC collection was less likely to be performed for female patients than male patients. This result is somewhat consistent with an analysis by Henning et al. which showed that the volume of blood collected for BCs was significantly greater for male patients than for female patients [24]. Furthermore, gender differences in sepsis management have been described. For example, Shallcross et al. found that male patients were more likely to receive BCs and antibiotics than female patients [13]. As has been consistently reported, men have a higher frequency of sepsis than women [25–27]. This distribution by gender may influence the decision of HCWs to administer adequate BC sampling more frequently to men.

Sensitivity analysis showed that specific infection sites had no influence on adequate BC sampling. We found that an unknown focus of infection increased the likelihood of adequate BC diagnostics. This could be due to the intense sufficient microbiological diagnostics needed to identify a pathogen. Furthermore, our analysis showed that a lack of documentation, especially of blood pressure, mental status, and infection foci, was an obstacle to general and adequate BC sampling. One explanation could be that these patients were in poor clinical condition and therefore required intensive treatment, which did not allow the documentation of clinical findings and BC sampling. However, it is also possible that organizational factors played a role. In critical care, as pointed out by Soto et al., disparities are most likely multifactorial, involving individual, community, and hospital-level factors [28]. Raupach-Rosin et al. found that the department in which a HCW worked was a factor associated with good BC practice, particularly for BC sampling [18]. Because we focused predominantly on patient factors that were associated with BC practice, we did not collect structural data from EDs that may influence BC practice. As shown by Pin et al., diagnostic stewardship training is not offered in every ED and should therefore be intensified [29].

Because we did not collect information on the treatment teams in the EDs, we were not able to describe the potential influence of gender-specific variations in practice on the part of physicians—there may, for example, have been greater adherence to guidelines among female physicians—that could also have resulted in differences in adequate blood culturing [30].

As mentioned above, a major limitation of our study is that our retrospective cohort was based on hospitals’ coding data of sepsis and using the ICD-10 code algorithm may underestimate the true incidence of sepsis [31]. Furthermore, as described by Schwarzkopf et al., accuracy of diagnosing and coding of sepsis varies between hospitals, which may have affected our analysis and biased the results [23]. Finally, all data was obtained retrospectively from patient charts and laboratory analysis of microbiological BCs. This method showed that relevant clinical information was not always documented. Therefore, we cannot precisely analyze clinical signs or the timing of AT administration in these cases. It could not be determined under which conditions BCs were initiated and collected, by whom, or the volume of blood cultured. Furthermore, data from only three EDs was analyzed and hence the generalizability of results is limited.

## Conclusion

Nevertheless, our analysis provides insight into current practices related to the pre-analytical phase of BC diagnostics for ED patients suspected of sepsis. More teaching and implementation of best practice in diagnostic stewardship should be undertaken to close the gaps in initiating and undertaking adequate BC sampling. The variations in BC sampling identified among EDs should be further investigated.

### Supplementary Information

Below is the link to the electronic supplementary material.

Supplementary file1 (DOCX 27 KB)

Supplementary file2 (DOCX 13 KB)

## Data Availability

The datasets used and/or analyzed during the current study are available from the corresponding author upon reasonable request.

## Abbreviations

AB: Antibiotics
AMS: Antimicrobial stewardship
AT: Antibiotic therapy
BC: Blood culture
CI: Confidence interval
ED: Emergency department
GCS: Glasgow Coma Scale
HCW: Healthcare worker
ICD: International Statistical Classification of Diseases and Related Health Conditions
IQR: Interquartile range
OR: Odds ratio
SOFA: Sequential Organ Failure Assessment
SSC: Surviving Sepsis Campaign
