# Supplementary material for: Deficits in blood culture collection in the emergency department if sepsis is suspected: results of a retrospective cohort study
Source: Infection. 2024 Mar 5;52(4):1385–96. doi: 10.1007/s15010-024-02197-x (PMC11289221; doi:10.1007/s15010-024-02197-x)
Supplement: Supplementary file 1 — Supplementary file1 (DOCX 27 KB) [file 15010_2024_2197_MOESM1_ESM.docx]

**Supplement Table 1 Supplement: General characteristics, comorbidities, clinical and laboratory findings in respect of organ dysfunction of patients with suspected sepsis in the emergency departments A-C (n=1143)**

| Parameter | Category | All | ED A | ED B | ED C | p-value |
| --- | --- | --- | --- | --- | --- | --- |
|  |  | n (%) / Median (IQR) | n (%) / Median (IQR) | | |  |
| Patients |  | 1,143 (100) | 112 (100) | 376 (100) | 655 (100) |  |
| Age (years) |  | 75 (65-81) | 79 (73.5-87.5) | 75 (64-81) | 74 (64-81) | <0.001 |
| Age (years) | < 65 | 280 (24.5) | 12 (10.7) | 95 (25.3) | 173 (26.4) | <0.001 |
|  | 65 - 74 | 271 (23.7) | 20 (17.9) | 85 (22.6) | 166 (25.3) |  |
|  | 75 - 84 | 412 (36) | 46 (41.1) | 138 (36.7) | 228 (34.8) |  |
|  | ≥ 85 | 180 (15.7) | 34 (30.4) | 58 (15.4) | 88 (13.4) |  |
| Gender | Male | 676 (59.1) | 60 (53.6) | 218 (58) | 398 (60.8) | 0.3605 |
|  | Female | 466 (40.8) | 52 (46.4) | 157 (41.8) | 257 (39.2) |  |
|  | Other | 1 (0.1) | 0 (0) | 1 (0.3) | 0 (0) |  |
| Documented comorbidities^A^ |  |  |  |  |  |  |
| · Chronic renal failure |  | 199 (17.4) | 26 (23.2) | 42 (11.2) | 131 (20) | 0.0004 |
| · Diabetes mellitus |  | 299 (26.2) | 39 (34.8) | 99 (26.3) | 161 (24.6) | 0.0742 |
| · Lymphoma |  | 24 (2.1) | 0 (0) | 0 (0) | 24 (3.7) | 0.0001 |
| · Leucemia |  | 17 (1.5) | 1 (0.9) | 0 (0) | 16 (2.4) | 0.0066 |
| · HIV/AIDS |  | 23 (2) | 0 (0) | 21 (5.6) | 2 (0.3) | <0.001 |
| Documented immunosupression^A^ |  | 542 (47.4) | 54 (48.2) | 156 (41.5) | 332 (50.7) | 0.0171 |
| ·  due to medication |  | 155 (13.6) | 2 (1.8) | 35 (9.3) | 118 (18) | <0.001 |
| ·  due to comorbidity |  | 466 (40.8) | 52 (46.4) | 145 (38.6) | 269 (41.1) | 0.0002 |
| Heart or pulse rate (beats/min) | ≥ 90 | 668 (58.4) | 71 (63.4) | 187 (49.7) | 410 (62.6) | <0.001 |
|  | < 90 | 296 (25.9) | 39 (34.8) | 77 (20.5) | 180 (27.5) |  |
|  | No information | 179 (15.7) | 2 (1.8) | 112 (29.8) | 65 (9.9) |  |
| Systolic blood pressure (mmHg) | ≤ 100 | 228 (19.9) | 23 (20.5) | 64 (17) | 141 (21.5) | <0.001 |
|  | 101 -< 140 | 481 (42.1) | 53 (47.3) | 113 (30.1) | 315 (48.1) |  |
|  | ≥ 140 | 260 (22.7) | 34 (30.4) | 86 (22.9) | 140 (21.4) |  |
|  | No information | 174 (15.2) | 2 (1.8) | 113 (30.1) | 59 (9) |  |
| Respiratory rate (breaths/min) | ≥ 20 | 324 (28.3) | 24 (21.4) | 39 (10.4) | 261 (39.8) | <0.001 |
|  | < 20 | 330 (28.9) | 76 (67.9) | 39 (10.4) | 215 (32.8) |  |
|  | No information | 489 (42.8) | 12 (10.7) | 298 (79.3) | 179 (27.3) |  |
| Body temperature (°C) | ≥ 38 | 522 (45.7) | 53 (47.3) | 176 (46.8) | 293 (44.7) | <0.001 |
|  | 36° < 38 | 408 (35.7) | 44 (39.3) | 97 (25.8) | 267 (40.8) |  |
|  | < 36 | 60 (5.2) | 9 (8) | 13 (3.5) | 38 (5.8) |  |
|  | No information | 153 (13.4) | 6 (5.4) | 90 (23.9) | 57 (8.7) |  |
| Suspected focus of infection | Respiratory tract | 228 (19.9) | 30 (26.8) | 46 (12.2) | 152 (23.2) | <0.001 |
|  | Urinary tract | 389 (34) | 34 (30.4) | 160 (42.6) | 195 (29.8) |  |
|  | Intraabdominal | 96 (8.4) | 11 (9.8) | 29 (7.7) | 56 (8.5) |  |
|  | Skin and soft tissue | 35 (3.1) | 3 (2.7) | 12 (3.2) | 20 (3.1) |  |
|  | Intravascular device | 11 (1) | 0 (0) | 1 (0.3) | 10 (1.5) |  |
|  | Other | 9 (0.8) | 0 (0) | 3 (0.8) | 6 (0.9) |  |
|  | Unknown | 239 (20.9) | 26 (23.2) | 88 (23.4) | 125 (19.1) |  |
|  | No information | 136 (11.9) | 8 (7.1) | 37 (9.8) | 91 (13.9) |  |
| Encephalopathy |  |  |  |  |  |  |
| Disorientation | Yes | 199 (17.4) | 30 (26.8) | 71 (18.9) | 98 (15) | 0.0022 |
|  | No | 477 (41.7) | 53 (47.3) | 147 (39.1) | 277 (42.3) |  |
|  | No information | 467 (40.9) | 29 (25.9) | 158 (42) | 280 (42.7) |  |
| Altered mentation^B^ | Yes | 229 (20) | 24 (21.4) | 57 (15.2) | 148 (22.6) | 0.0001 |
|  | No | 663 (58) | 80 (71.4) | 222 (59) | 361 (55.1) |  |
|  | No information | 251 (22) | 8 (7.1) | 97 (25.8) | 146 (22,3) |  |
| Respiratory dysfunction^C^ | Yes | 393 (34.4) | 62 (55.4) | 102 (27.1) | 229 (35) | <0.001 |
|  | No | 553 (48.4) | 47 (42) | 141 (37.5) | 365 (55.7) |  |
|  | No information | 197 (17.2) | 3 (2.7) | 133 (35.4) | 61 (9.3) |  |
| Renal dysfunction^D^ | Yes | 343 (30) | 30 (26.8) | 108 (28.7) | 205 (31.3) | <0.001 |
|  | No | 769 (67.3) | 80 (71.4) | 243 (64.6) | 446 (68.1) |  |
|  | No information | 31 (2.7) | 2 (1.8) | 25 (6.6) | 4 (0.6) |  |
| Liver failure^E^ | Yes | 166 (14.5) | 14 (12.5) | 44 (11.7) | 108 (16.5) | <0.001 |
|  | No | 618 (54.1) | 45 (40.2) | 137 (36.4) | 436 (66.6) |  |
|  | No information | 359 (31.4) | 53 (47.3) | 195 (51.9) | 111 (16.9) |  |
| Coagulation failure^F^ | Yes | 451 (39.5) | 41 (36.6) | 161 (42.8) | 249 (38) | <0.001 |
|  | No | 630 (55.1) | 65 (58) | 176 (46.8) | 389 (59.4) |  |
|  | No information | 62 (5.4) | 6 (5.4) | 39 (10.4) | 17 (2.6) |  |
| Elevated INR^G^ | Yes | 494 (43.2) | 50 (44.6) | 140 (37.2) | 304 (46.4) | 0.003 |
|  | No | 126 (11) | 15 (13.4) | 33 (8.8) | 78 (11.9) |  |
|  | No information | 523 (45.8) | 47 (42) | 203 (54) | 273 (41.7) |  |
| Thrombocytopenia^H^ | Yes | 266 (23.3) | 27 (24.1) | 64 (17) | 175 (26.7) | <0.001 |
|  | No | 361 (31.6) | 36 (32.1) | 112 (29.8) | 213 (32.5) |  |
|  | No information | 516 (45.1) | 49 (43.8) | 200 (53.2) | 267 (40.8) |  |
| Elevated lactate level^I^ | Yes | 512 (44.8) | 48 (42.9) | 133 (35.4) | 331 (50.5) | <0.001 |
|  | No | 352 (30.8) | 21 (18.8) | 89 (23.7) | 242 (36.9) |  |
|  | No information | 279 (24.4) | 43 (38.4) | 154 (41) | 82 (12.5) |  |
| Total leukocyte count | Leukocytosis^J^ | 682 (59.7) | 73 (65.2) | 252 (67) | 357 (54.5) | <0.001 |
|  | Leukopenia^K^ | 98 (8.6) | 4 (3.6) | 16 (4.3) | 78 (11.9) |  |
|  | Normal leukocyte count^L^ | 356 (31.1) | 33 (29.5) | 107 (28.5) | 216 (33) |  |
|  | No information | 7 (0.6) | 2 (1.8) | 1 (0.3) | 4 (0.6) |  |

ED: Emergency department; IQR: Interquartile range

^A^Multiple answers possible

^B^Altered mentation: GCS (Glasgow Coma Scale) < 15

^C^Respiratory dysfunction: Oxygen saturation < 95%

^D^Renal dysfunction^:^ Elevated serum creatinine level ≥ 1.3 mg/dl male, ≥ 1.1mg/dl female

^E^Liver failure: Elevated bilirubin level ≥ 1.2 mg/dl

^F^Coagulation failure: INR (international normalized ratio) > 1.25 and/or Thrombocytopenia < 150 x10^9^/L

^G^Pathological INR: > 1.25

^H^Thrombocytopenia: < 150 x10^9^/L

^I^Elevated lactate level: > 18.0 mg/dl

^J^Normal white blood count: 4-12 (x10^9^/L)

^K^Leukocytosis: ≥ 12 (x10^9^/L)

^L^Leukopenia: < 4 (x10^9^/L)
