# Supplementary material for: Deficits in blood culture collection in the emergency department if sepsis is suspected: results of a retrospective cohort study
Source: Infection. 2024 Mar 5;52(4):1385–96. doi: 10.1007/s15010-024-02197-x (PMC11289221; doi:10.1007/s15010-024-02197-x)
Supplement: Supplementary file 2 — Supplementary file2 (DOCX 13 KB) [file 15010_2024_2197_MOESM2_ESM.docx]

**Supplement table 2: Positivity rate in accordance to recommended time sequence of BC sampling and administration of antibiotic therapy (n=946 patients)**

| Parameter | Category | BC sampling prior to AT administration  (n=704) | | BC sampling after AT administration  (n=242) | | p-value |
| --- | --- | --- | --- | --- | --- | --- |
|  |  | n | % | n | % |  |
| Pathogen(s) identified in all patients | No | 265 | 37.6 | 111 | 45.9 | 0.024 |
|  | Yes | 439 | **62.4** | 131 | **54.1** |  |
| Pathogen(s) identified in patients receiving 1 BC set | No | 145 | 40.8 | 77 | 46.7 | 0.212 |
|  | Yes | 210 | **59.2^A^** | 88 | **53.3^B^** |  |
| Pathogen(s) identified in patients receiving ≥2 BC sets | No | 120 | 34.4 | 34 | 44.2 | 0.106 |
|  | Yes | 229 | **65.6^A^** | 43 | **55.8^B^** |  |

BC: Blood culture; AT: Antibiotic therapy

^A^ p<0.001

**^B^** p=0.715
